# Genetic Resistance to Avian Leukosis Viruses Induced by CRISPR/Cas9 Editing of Specific Receptor Genes in Chicken Cells

**DOI:** 10.3390/v10110605

**Published:** 2018-11-02

**Authors:** Anna Koslová, Dana Kučerová, Markéta Reinišová, Josef Geryk, Pavel Trefil, Jiří Hejnar

**Affiliations:** 1 Institute of Molecular Genetics, Czech Academy of Sciences, Videnska 1083, CZ 14220 Prague 4, Czech Republic; anna.koslova@mpimf-heidelberg.mpg.de (A.K.); dana.kucerova@img.cas.cz (D.K.); marketa.reinisova@img.cas.cz (M.R.); geryk@img.cas.cz (J.G.); 2 BIOPHARM, Research Institute of Biopharmacy and Veterinary Drugs, 254 49 Jílové u Prahy, Czech Republic; trefil@bri.cz

**Keywords:** avian leukosis virus, retrovirus receptor, virus-resistance in chicken, CRISPR/Cas9

## Abstract

Avian leukosis viruses (ALVs), which are pathogens of concern in domestic poultry, utilize specific receptor proteins for cell entry that are both necessary and sufficient for host susceptibility to a given ALV subgroup. This unequivocal relationship offers receptors as suitable targets of selection and biotechnological manipulation with the aim of obtaining virus-resistant poultry. This approach is further supported by the existence of natural knock-outs of receptor genes that segregate in inbred lines of chickens. We used CRISPR/Cas9 genome editing tools to introduce frame-shifting indel mutations into *tva*, *tvc*, and *tvj* loci encoding receptors for the A, C, and J ALV subgroups, respectively. For all three loci, the homozygous frame-shifting indels generating premature stop codons induced phenotypes which were fully resistant to the virus of respective subgroup. In the *tvj* locus, we also obtained in-frame deletions corroborating the importance of W38 and the four amino-acids preceding it. We demonstrate that CRISPR/Cas9-mediated knock-out or the fine editing of ALV receptor genes might be the first step in the development of virus-resistant chickens.

## 1. Introduction

Avian leukosis viruses (ALV) have been recognized as the cause of serious commercial losses in poultry industry since the 1920s. Among the six phylogenetically relative subgroups (A, B, C, D, E, and J) present in domestic chickens, ALV-J remains in broiler flocks and even spreads into layer breeds in China and other Asian countries [1]. A new challenge to the poultry industry might come with the emergence of new pathogenic ALV strains, as either mutants or recombinants with endogenous counterparts [2]. The ALV subgroups have traditionally been classified by their range of susceptible/resistant hosts, antibody cross-neutralization, and interference in superinfection experiments [3].

The discovery of subgroup-specific receptors has explained the molecular mechanism of genetic susceptibility to ALVs [4]. Tva, a protein of the low-density lipoprotein receptor family [5,6], serves as the receptor for the ALV-A subgroup. Subgroups B, D, and E utilize the same Tvb receptor, a tumor necrosis factor receptor-related protein [7,8,9]. Subgroup C ASLVs enters cells via the Tvc protein of the butyrophilin family, which consists two immunoglobulin-like domains [10]. Individually, Tva, Tvb, and Tvc each have unique transmembrane domains, whereas Tvj, the subgroup J receptor, possesses 12 predicted transmembrane segments that together with extra- and intracellular domains build the channel structure of the chicken Na^+^/H^+^ exchanger type 1 (chNHE1) [11].

There is an unequivocal relationship between the cell susceptibility to the given ALV subgroup and the display of the respective intact receptor on the cell surface. For example, the cell susceptibility to ALV-C can be conferred in resistant mammalian cells by the ectopic expression of the *tvc* receptor gene and, vice versa, genetic knock-out of *tvc* abrogates host susceptibility in DT-40 cells [10]. These experiments formally demonstrate that these receptors are both necessary and sufficient for cell susceptibility and that no co-receptors play a role in ALV entry.

In *tva*, *tvb*, and *tvc* receptor loci, there are virus-resistant alleles that bear either a frame-shift mutation or exhibit the substitution of critical cysteine residues [10,12,13]. These virus-resistant alleles segregate in inbred lines of domestic chickens, which are thus resistant to the respective subgroup of ALV. All inbred lines and breeds of domestic chicken are susceptible to ALV-J but most of galliform birds which are closely related to domestic chickens are resistant due to the deletion of single amino-acid, tryptophan W38, of NHE1 [14,15,16]. Another source of mutations that affect the receptor function are chicken lines with decreased susceptibility to ALVs. We have previously described the substitution of subcritical cysteine residue in *tvb* [17] and polymorphic intronic deletions in *tva*, which strongly reduce Tva receptor expression and display [18].

There have been no visible side effects of virus-resistant alleles on the animal health or reproduction detected, hence we can assume that such alleles could be biotechnologically introduced also into commercial breeds of poultry. The first attempt to genetically knock-out the ALV receptor locus was demonstrated [19,20] using the CRISPR/Cas9 targeting chicken *tvb* and *tvj*, respectively. In the present study, we have shown that all remaining ALV receptor loci can be targeted using CRISPR/Cas9 technology resulting in resistance to the respective ALV subgroups.

## 2. Materials and Methods

### 2.1. Construction of the Gene Editing CRISPR/Cas9 Vectors

**Figure 1:**
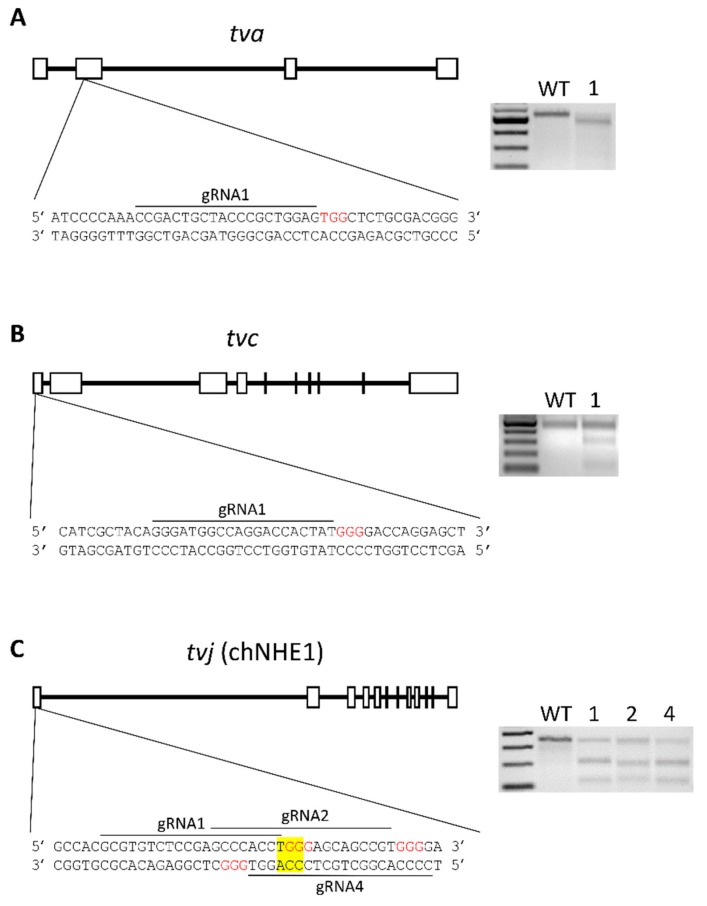
Design of gRNAs of gene editing CRISPR/Cas9 constructs for chicken avian leukosis virus (ALV) receptor genes *tva* (**A**), *tvc* (**B**), and *tvj* (**C**) and their indel generation activity in DF-1 cells. Exon-intron gene structures, the gRNA complementary sequences (underlined), and protospacer adjacent motifs (red) are shown in the left part. TGG trinucleotide coding for the critical W38 of chNHE1 is highlighted in yellow. Results of the T7EI assay for each of the CRISPR/Cas9 constructs are shown as agarose electrophoresis controlled by markers of molecular size and wild-type DF-1 cells (right).

We used CRISPR/Cas9 genome editing tools and cloned the guide RNA sequences that matched the coding sequences of chicken *tva*, *tvc*, and *tvj* genes into the sgRNA scaffold of PX458 vector which is available as the AddGene vector pSpCas9BB-2A-GFP, number 48138 [21]. We edited both the *tva* and *tvc* genes using single guide RNAs (gRNA) and constructed three vectors with different gRNAs for *tvj*. The CRISPR design tool (http://crispr.mit.edu) [22] was used to identify candidate gRNA sequences in the first or second exon of *tva*, *tvc*, or *tvj*. The gRNA sequences and their match to the respective receptor genes are shown in Figure 1. The DNA sequences encoding the gRNAs (Table 1) were synthesized as sense and anti-sense oligonucleotides (Eurogentec, Liege, Belgium). The matching pairs were mixed, 5′ phosporylated by T4 polynucleotide kinase (New England Biolabs, Ipswich, MA, USA), denaturated for 5 min at 95 °C, and annealed by slow cooling (−0.1 °C/s) to 25 °C. The PX458 vector was cleaved by BbsI and annealed oligonucleotides were ligated by Quick ligase (New England Biolabs) to form pX458-TVA, pX458TVC, and pX458-TVJ1/2/4.

### 2.2. DF-1 Cell Culture and Genome Editing

The chicken cell line DF-1 [23] was grown in a mixture of two parts Dulbecco’s modified Eagle’s medium and 1 part F-12 medium supplemented with 8% fetal calf serum, 2% chicken serum, and 1× antibiotic-antimycotic solution (Sigma, St. Louis, MO, USA) under 5% CO_2_ atmosphere at 37 °C. DF-1 cells were transfected with CRISPR/Cas9 vectors using Lipofectamine 3000 (Thermo Fisher Scientific, Waltham, MA, USA) according to the manufacturer’s protocol. Three days post transfection, we observed approximately 10–30% GFP-positive cells. We then collected 10,000 cells for each CRISPR/Cas9 vector to determine the efficiency of each gRNA in the T7E1 assay. In parallel, we sorted the cells with the highest GFP expression into 96-well tissue culture plates using the Influx cell sorter (Becton-Dickinson, Franklin Lakes, NJ, USA) in single-cell sort mode. The resulting single-cell clones were then used for analysis of CRISPR/Cas9-introduced mutations.

### 2.3. Determining Genome Targeting Efficiency Using the T7EI Assay

The aliquots of DF-1 cells transfected with CRISPR/Cas9 vectors were collected (as described in Section 2.2), subcultured, and harvested for genomic DNA extraction. The targeted regions of *tva*, *tvc*, and *tvj* genes were amplified using the primers TVA-fw and TVA-rv for *tva*, TVC-fw and TVC-rv for *tvc*, and TVJ-fw and TVJ-rv for *tvj* (primer sequences listed in Table 1). The cycling conditions were as follows: 98 °C for 3 min, 40 cycles of 10 s at 98 °C, 30 s annealing, and 30 s amplification at 72 °C. The final amplification was held for 5 min. The annealing temperatures were 65 °C for *tva* and *tvj* and 61 °C for *tvc*. TaKaRa Taq DNA polymerase (TaKaRa, Kusacu, Japan) was used for for *tva* and *tvc* amplification while TaKaRa Ex Taq HS DNA polymerase was used for *tvj* amplification. The T7E1 assay was used to determine indel efficiency. Briefly, 200 ng of the resulting PCR products were denatured in 19 μL 1× NEB buffer 2 for 5 min at 95 °C, quickly chilled to 85 °C (−2 °C/s) and reannealed by slowly decreasing the temperature (−0.1 °C/s) from 85 °C to 25 °C. Then, 10 U of T7 Endonuclease I (New England Biolabs, Ipswich, MA, USA) were added for 20 min at 37 °C. Cleavage of heteroduplex amplicons was then analyzed by agarose electrophoresis.

### 2.4. Analysis of Gene-Edited Single-Cell Clones of DF-1 Cells

DF-1 single-cell clones were expanded from the previously sorted GFP-positive cells (see Section 2.2) and subcultured by passaging 25% of the cells three times a week. Genomic DNA was extracted and the presence of indel mutations in *tva*, *tvc*, and *tvj* receptor genes, either in heterozygous or homozygous state, was determined by PCR amplification of the target region (see Section 2.3). The presence of wt and/or shortened PCR fragments indicated the intact and/or edited receptor alleles. From a representative number of cell clones, particularly the virus resistant ones, the status of receptor loci was confirmed by capillary DNA sequencing.

### 2.5. Viral Propagation and Cell Infection

Infectious GFP reporter-transducing viruses for susceptibility/resistance assays were produced in DF-1 cells which were transfected with RCASBP(A)GFP, RCASBP(C)GFP [24], or RCASBP(J)GFP [14] plasmid DNA. Virus stocks were harvested on day 9 or 10 post transfection. The cell supernatants were cleared of debris by centrifugation at 2000× *g* for 10 min at 10 °C and aliquoted viral stocks were stored at −80 °C. The virus titer was determined by terminal dilution of virus stock and subsequent infection of DF-1 cells which reached 10^6^ infection units (IU) per ml. Susceptibility to the respective ALV subgroup was assessed by viral spread as described previously [17]. Briefly, DF-1 cell clones were seeded at a density of 5 × 10^4^ per well in a 24-well plate and infected with RCASBP(A)GFP, RCASBP(C)GFP, or RCASBP(J)GFP virus at a multiplicity of infection of 10 the day after seeding. Infected cells were inspected by inverse fluorescence microscope Leica DM IRB (Leica, Wetzlar, Germany) and percentage of GFP-positive cells was quantitated by fluorescence-activated cell sorting (FACS) using an LSR II analyzer (Becton, Dickinson, Franklin Lakes, NJ, USA) on day 3 post infection. The cells were trypsinized, washed in phosphate buffered saline (PBS), and resuspended in Hoechst solution (Sigma, St. Louis, MO, USA) before analysis. Cells that exhibited less than 0.05% of GFP-positivity were regarded resistant to the virus of the respective subgroup. We did not observe any cytotoxicity after infection with the three RCAS vectors used in this study.

## 3. Results

### 3.1. Design of Gene Editing Experiments

In order to introduce the disrupting mutations into the ALV receptor genes *tva*, *tvc*, and *tvj*, we constructed respective CRISPR/Cas9 vectors with a GFP reporter gene and transfected them transiently into DF-1 cells. The design of guide RNA sequences and their correspondence to the respective receptor genes are shown in Figure 1. The efficiency of CRISPR/Cas9 target cleavage was tested in the T7 endonuclease I assay. GFP-positive single cell clones were isolated and expanded from cultures containing high amount of mismatches in target locus. The expanded cell clones were challenged with the respective subgroup of RCAS vectors, RCASBP(A)GFP, RCASBP(C)GFP, or RCASBP(J)GFP and the susceptibility/resistance was examined as a percentage of GFP-positive cells. Tva, *tvc*, or *tvj* editing was tested by PCR and clones with homozygous mutations were selected.

### 3.2. Gene Editing of tva Gene Confers the Resistance to ALV Subgroup A

**Figure 2:**
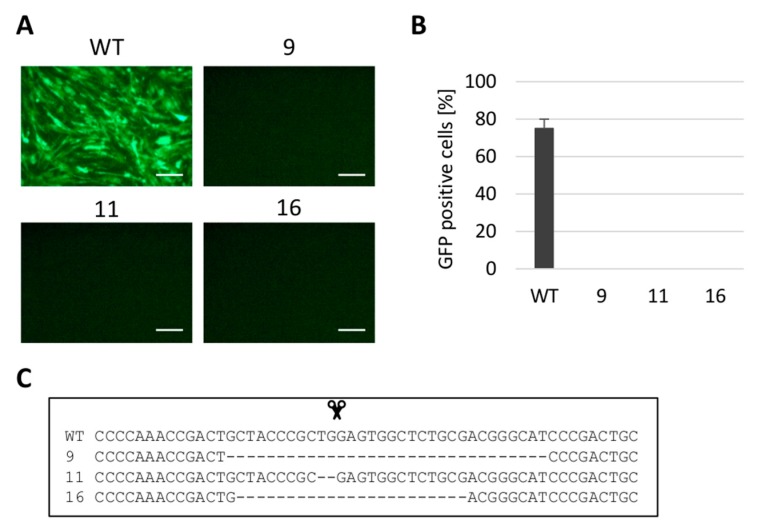
Analysis of ALV-A-resistant clones of DF-1 cells. The selected clones 9, 11, and 16 were challenged with the RCASBP(A)GFP reporter virus. The virus spread in the cell culture was assayed as the presence of GFP-positive cells by fluorescence microscopy (**A**) or FACS (**B**). The homozygous deletion mutations of the *tva* gene present in these three clones are shown (**C**) with the cleavage point represented by the scissors. The FACS experiment was done in two parallels in two independent experiments and the results are shown as the mean ± standard errors. Scale bar = 100 μm.

We established 20 single cell clones in the *tva* gene editing experiment, in which the second exon was targeted with one gRNA (Figure 1). In 15 of the clones, the challenge with the RCASBP(A)GFP reporter virus resulted in the resistance to the A subgroup ALV. The sequencing analysis showed homozygous deletions of 32, 2, and 23 bp around the cleavage site in clones No. 9, 11, and 16, respectively. The resistance to RCASBP(A)GFP and deletion mutations in these three clones are given in Figure 2. In an additional 12 virus-resistant clones, we found different indel mutations in both *tva* alleles (data not shown). As expected, we observed at least one intact *tva* allele as a wild type (wt)-sized PCR fragment in each of the five RCASBP(A)GFP-susceptible clones. These results clearly demonstrate the dependence of subgroup A virus entry on the intact *tva* sequence and the possibility to efficiently generate resistant genotypes using CRISPR/Cas9 techniques.

### 3.3. Gene Editing of tvc Gene Renders the Cells Resistant to ALV Subgroup C

**Figure 3:**
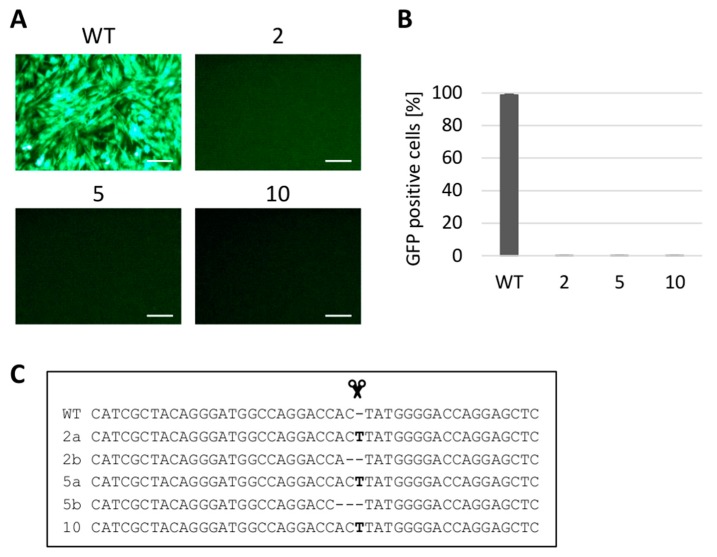
Analysis of ALV-C-resistant clones of DF-1 cells. The selected clones 2, 5, and 10 were challenged with the RCASBP(C)GFP reporter virus. The virus spread in the cell culture was assayed as the presence of GFP-positive cells by fluorescence microscopy (**A**) or FACS (**B**). The indel mutations present in both *tvc* alleles of these three clones are shown (**C**) with the cleavage point represented by the scissors. Insertions of T nucleotides are shown as bold letters. The FACS experiment was done in two parallels in two independent experiments and the results are shown as the mean + standard errors. Scale bar = 100 μm.

In two *tvc* gene editing experiments, we targeted the first coding exon with one gRNA and established 12 single-cell clones including five clones which were fully resistant to the RCASBP(C)GFP reporter virus. In Figure 3, the virus challenge results and the respective *tvc* sequences are shown for the resistant clone numbers 2, 5, and 10. The gene editing of the *tvc* locus turned out to be less efficient than that of *tva* locus, and only one clone (number 10) displayed homozygous insertion of the T nucleotide at the CRISPR/Cas9 cleavage site. The sequencing analysis of two resistant clones, numbers 2 and 5, showed only heterozygous indel mutations: the T insertion together with either 1 or 2 bp deletions in clones 2 and 5, respectively. In seven virus-susceptible clones, we found different indel mutations, but always in only one allele of the *tvc* locus, as indicated by the presence of wt and shortened PCR fragments (data not shown). Thus, similarly to the *tva*, we again corroborated the dependence of subgroup C virus entry on the intact *tvc* sequence. Although the gene editing was less efficient at the *tvc* locus, the creation of resistant genotypes was feasible using the CRISPR/Cas9 technique.

### 3.4. Gene Editing of chNHE1 (tvj) Renders the Cells Resistant to ALV Subgroup J

**Figure 4:**
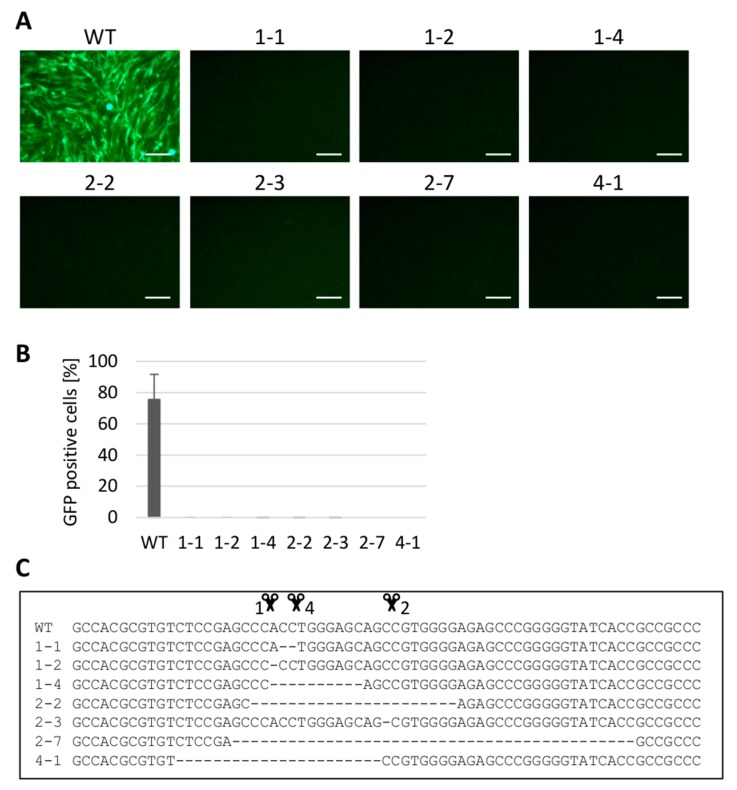
Analysis of *chNHE1*-editied clones of DF-1 cells containing frameshifting deletions. The selected clones were challenged with the RCASBP(J)GFP reporter virus. The virus spread in the cell culture was assayed as the presence of GFP-positive cells by fluorescence microscopy (**A**) or FACS (**B**). The deletions present in both *chNHE1* alleles of these clones are shown (**C**) with the cleavage points represented by the scissors. The FACS experiment was done in two parallels in two independent experiments and the results are shown as the mean ± standard errors. Scale bar = 100 μm.

**Figure 5:**
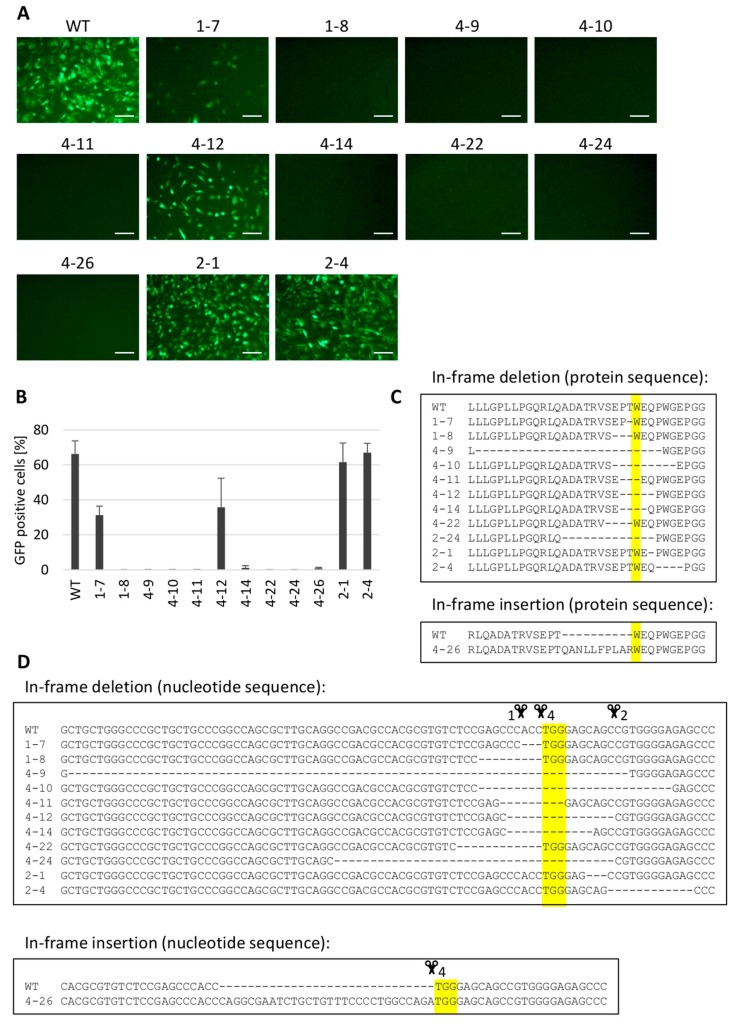
Analysis of *chNHE1*-editied clones of DF-1 cells containing in-frame mutations. The selected clones were challenged with the RCASBP(J)GFP reporter virus. The virus spread in the cell culture was assayed as the presence of GFP-positive cells by fluorescence microscopy (**A**) or FACS (**B**). Predicted amino-acid sequence of chNHE1 (position 15-47) in selected clones is depicted (**C**). The in-frame deletions and one in-frame insertion homozygous in both *chNHE1* alleles of these clones are shown (**D**) with the cleavage points represented by the scissors. The critical W38 and the corresponding TGG trinucleotide are highlighted in yellow. The FACS experiment was done in two parallels in two independent experiments and the results are shown as the mean ± standard errors. Scale bar = 100 μm.

The chNHE1 is a particularly important target for CRISPR/Cas9-mediated genome editing because it is critical for the entry of ALV-J into the cell, which is commonly found in commercial breeds of chicken. The precise deletion of TGG nucleotides that code for W38 could abrogate the receptor activity and retain the Na^+^/H^+^ exchanger function of the chNHE1 molecule. We, therefore, designed three different gRNAs to target the sequence encoding W38 (Figure 1C) and applied the respective PX458 constructs to edit the *chNHE1* gene. We analyzed by DNA sequencing 9, 9, and 35 clones obtained using the CRISPR/Cas9 constructs with gRNA1, gRNA2 and gRNA4, respectively. All three CRISPR/Cas9 constructs proved to be very efficient with more than 90% of clones containing homozygous deletions in the target region. We present separately the clones bearing homozygous frameshift deletions in the cleavage region of *chNHE1* (Figure 4) and clones with homozygous in-frame indel mutations (Figure 5).

The homozygous frameshift deletions in *chNHE1* of selected clones are shown in Figure 4C. All tested clones were fully resistant to the RCASBP(J)GFP reporter virus (Figure 4A,B). The clones with in-frame indel mutations introducing the deletion or insertion of one or several amino-acids in the first extracellular loop (ECL1) of chNHE1 (exemplified in Figure 5C,D) provided more complex results when challenged with RCASBP(J)GFP virus (Figure 5A,B). Clones with deletions involving W38 (clones 4-9, 4-10, 4-11, 4-14, 4-22) turned out to be resistant to virus infection, with only one exception, clone 4-12 which contained a deletion of five amino-acids. We hypothesize that in this case, W42 is partially able to replace W38. Also deletions which do not involve W38 displayed a significant impact on cell susceptibility to virus infection; e.g., the deletions of three or four amino-acids before W38 (clones 1-8, 4-22) conferred full resistance to the RCASBP(J)GFP virus. On the contrary, deletion of single amino-acid T37 (clone 1-7) had only a mild effect and the deletions which were introduced behind W38 (clones 2-1 and 2-4) did not affect cell susceptibility to RCASBP(J)GFP at all. In-frame insertions around W38 deletion can have a similar deleterious effect on the chNHE1 receptor activity just as in the case of deletions. We found large insertion of 10 amino-acids before W38 (clone 4-26), which resulted in complete resistance to virus infection. These results clearly demonstrate that ALV-J strongly depends on the intact chNHE1 receptor and confirm the crucial role of W38. Furthermore, we suggest that resistant genotypes could be generated using CRISPR/Cas9 techniques.

## 4. Discussion

In summary, our results clearly demonstrate the dependence of virus entry on intact sequences of respective receptors for the ALV subgroups A, C, and J. The frame-shifting indel mutations introduced into the *tva*, *tvc*, and *chNHE1* (*tvj*) genes using the CRISPR/Cas9 gene editing system conferred resistance to ALV subgroups A, C, and J, respectively, in clones of chicken DF-1 cells. Although this unequivocal dependence of ALV entry on the display of specific cell receptors was already shown in *tvc^−/−^* DT40 cells [10], which maintain homologous recombination, the CRISPR/Cas9 system allows gene editing in all types of cells including primordial germ cells (PGCs). The targeting of PGCs is a prerequisite for the genetic in vivo knock-out in chicken [25] and it was recently published that the orthotopic transplantation of PGC into adult roosters [26] increased the likelihood of generating ALV-resistant chicken lines using CRISPR/Cas9 techniques.

CRISPR/Cas9-mediated knock out of ALV receptor locus was previously demonstrated for chicken *tvb* [19]. This study was performed using CRISPR/Cas9 vectors containing puromycin resistance gene and transfected clones were selected for antibiotic resistance. In comparison with our experimental set-up, lower *tvb* targeting was observed among the puromycin-resistant clones. Surprisingly, introduction of premature stop codons into the *tvb* coding sequence did not lead to cell clones fully resistant to ALV-B, although the susceptibility was significantly decreased. It remains to be understood if the priming of virus envelope glycoproteins by the short soluble receptor fragment could play a role.

The strategy of CRISPR/Cas9 editing of virus receptor has already been applied in pigs, where indel mutations of CD163 made the gene-edited pigs resistant to both porcine reproductive and respiratory syndrome viruses [27]. A genome-wide association study in pigs revealed only one genomic region that showed a significant response to this virus [28], which limits the marker-assisted selection for natural resistance. In the case of ALV-J in chicken, currently no genetic sources of heritable resistance are known and no mutations of the critical region of the *chNHE1* gene have been found [15]. In both instances, the biotechnology approach to the resistance might be an alternative.

Although the *tva*, *tvb*, and *tvc* virus-resistant alleles with either frame-shift mutations or inactivating cysteine residue substitutions exist in inbred lines of domestic chicken [10,12,13], no additional phenotypes have been described and the primary physiological functions of these loci remain to be explored. This is, however, not the case of chNHE1, which has been shown to have a clearly defined Na^+^/H^+^ exchange function and integrated roles in regulation of cellular pH, osmotic activity, size, shape, adhesion, migration and proliferation rate [29]. We did not observe any side effects of our chNHE1 inactivating indel mutations in either cell culture or flow cytometry, during the expansion of cell clones and subsequent long-term passage of cells. On the other hand, a previous report described remodeling of actin cytoskeleton and gross effects on focal adhesions, cell polarity, and chemotactic motility [30]. The importance of NHE1 is also visible in tumors, where oncogene-activated NHE1 promotes resorption of extracellular matrix and mesenchymal migration of tumor cells [31], and, vice versa, inhibition of NHE1 reduces cell invasion capacity [32]. 

In regards to an in vivo application, the precise gene editing of virus-binding determinants in chNHE1 is an option. Fortunately, the critical amino-acid residue, W38, resides in the first extracellular loop (ECL1) of chNHE1 [14]. ECL1 is the most prominent loop and shows a low degree of conservation among birds [15]. It has been shown that proteolytic cleavage of ECL1 does not affect NHE1 activity [33] and, additionally, the elimination of N- and O-linked glycosylation sites is not crucial for Na^+^/H^+^ exchange or protein display [34]. Furthermore, W38 deletions or substitutions are common in closely related galliform birds [14,16]. Recently, CRISPR/Cas9-mediated homologous recombination and W38 deletion was demonstrated [20]. Taken together, the precise gene editing of ECL1 in chicken PGC combined with the methods of chicken transgenesis might be the way toward a ALV-J-resistant chicken line.

Our chNHE1 indel mutations that were induced by the CRISPR/Cas9 system also suggest that W38 is not the only way to a ALV-J-resistant phenotype. Amino-acid residues preceding W38 could be of the same importance for the receptor function (see the clones 1-8, 4-22) and a more detailed analysis remains to be done. Residues 28 to 39 have been associated with ALV-J binding and entry [35]. On the other hand, some deletions including W38 must not confer the resistance to ALV-J because W41 could replace the missing W38 (see the clone 12). The detailed knowledge of critical amino-acid residues in ECL1 will provide us with new targets for the biotechnological development of ALV-J-resistant poultry.

## Figures and Tables

**Table 1:** Oligonucleotides used in this study.

Oligonucleotide Name	Sequence (5′–3′)
TVA-gRNA1a	CACCGCCGACTGCTACCCGCTGGAG
TVA-gRNA1b	AAACCTCCAGCGGGTAGCAGTCGGC
TVC-gRNA1a	CACCGGGATGGCCAGGACCACTATG
TVC-gRNA1b	AAACATAGTGGTCCTGGCCATCCCG
TVJ-gRNA1a	CACCGCGTGTCTCCGAGCCCACCT
TVJ-gRNA1b	AAACAGGTGGGCTCGGAGACACGC
TVJ-gRNA2a	CACCGCCCACCTGGGAGCAGCCGT
TVJ-gRNA2b	AAACACGGCTGCTCCCAGGTGGGC
TVJ-gRNA4a	CACCGCCCCACGGCTGCTCCCAGGT
TVJ-gRNA4b	AAACACCTGGGAGCAGCCGTGGGGC
TVA-fw	GCATGGTGCGGTTGTTGGAG
TVA-rv	CTGTGCCGCCGGCGGTGGGC
TVC-fw	CTCGCTGGCAGAGCCAGGAC
TVC-rv	CAAAATGTGGCCCTGATGAAGA
TVJ-fw	CGGCTCCCTCCGCCATG
TVJ-rv	TCATCAGGCAGGCCAGCAGGAT

## References

[1] Payne L.N., Nair V. (2012). The long view: 40 years of avian leukosis research. Avian Pathol..

[2] Cui N., Su S., Chen Z., Zhao X., Cui Z. (2014). Genomic sequence analysis and biological characteristics of a rescued clone of avian leukosis virus strain JS11C1, isolated from indigenous chickens. J. Gen. Virol..

[3] Weiss R.A., Levy J.A. (1992). Cellular receptors and viral glycoproteins involved in retrovirus entry. The Retroviridae.

[4] Barnard R.J.O., Elleder D., Young J.A.T. (2006). Avian sarcoma and leukosis virus-receptor interactions: From classical genetics to novel insights into virus-cell membrane fusion. Virology.

[5] Bates P., Young J.A.T., Varmus H.E. (1993). A receptor for subgroup A Rous sarcoma virus is related to the low density lipoprotein receptor. Cell.

[6] Young J.A.T., Bates P., Varmus H.E. (1993). Isolation of a chicken gene that confers susceptibility to infection by subgroup A avian leukosis and sarcoma viruses. J. Virol..

[7] Brojatsch J., Naughton J., Rolls M.M., Zingler K., Young J.A. (1996). CAR1, a TNFR-related protein, is a cellular receptor for cytopathic avian leukosis-sarcoma viruses and mediates apoptosis. Cell.

[8] Adkins H.B., Brojatsch J., Naughton J., Rolls M.M., Pesola J.M., Young J.A.T. (1997). Identification of a cellular receptor for subgroup E avian leukosis virus. Proc. Natl. Acad. Sci. USA.

[9] Adkins H.B., Brojatsch J., Young J.A.T. (2000). Identification and characterization of a shared TNFR-related receptor for subgroup B, D, and E avian leukosis viruses reveal cysteine residues required specifically for subgroup E viral entry. J. Virol..

[10] Elleder D., Stepanets V., Melder D.C., Šenigl F., Geryk J., Pajer P., Plachý J., Hejnar J., Svoboda J., Federspiel M.J. (2005). The receptor for the subgroup C avian sarcoma and leukosis viruses, Tvc, is related to mammalian butyrophilins, members of the immunoglobulin superfamily. J. Virol..

[11] Chai N., Bates P. (2006). Na/H exchanger type 1 is a receptor for pathogenic subgroup J avian leukosis virus. Proc. Natl. Acad. Sci. USA.

[12] Klucking S., Adkins H.B., Young J.A.T. (2002). Resistance to infection by subgroups B, D, and E avian sarcoma and leukosis viruses is explained by a premature stop codon within a resistance allele of the tvb receptor gene. J. Virol..

[13] Elleder D., Melder D.C., Trejbalová K., Svoboda J., Federspiel M.J. (2004). Two different molecular defects in the Tva receptor gene explain the resistance of two tvar lines of chickens to infection by subgroup A avian sarcoma and leukosis viruses. J. Virol..

[14] Kučerová D., Plachý J., Reinišová M., Šenigl F., Trejbalová K., Geryk J., Hejnar J. (2013). Nonconserved tryptophan 38 of the cell surface receptor for subgroup J avian leukosis virus discriminates sensitive from resistant avian species. J. Virol..

[15] Reinišová M., Plachý J., Kučerová D., Šenigl F., Vinkler M., Hejnar J. (2016). Genetic Diversity of NHE1, Receptor for Subgroup J Avian Leukosis Virus, in Domestic Chicken and Wild Anseriform Species. PLoS ONE.

[16] Plachý J., Reinišová M., Kučerová D., Šenigl F., Stepanets V., Hron T., Trejbalová K., Elleder D., Hejnar J. (2017). Identification of New World Quails Susceptible to Infection with Avian Leukosis Virus Subgroup J. J. Virol..

[17] Reinišová M., Šenigl F., Yin X., Plachý J., Geryk J., Elleder D., Svoboda J., Federspiel M.J., Hejnar J. (2008). A single-amino-acid substitution in the TvbS1 receptor results in decreased susceptibility to infection by avian sarcoma and leukosis virus subgroups B and D and resistance to infection by subgroup E in vitro and in vivo. J. Virol..

[18] Reinišová M., Plachý J., Trejbalová K., Šenigl F., Kučerová D., Geryk J., Svoboda J., Hejnar J. (2012). Intronic deletions that disrupt mRNA splicing of the *tva* receptor gene result in decreased susceptibility to infection by avian sarcoma and leukosis virus subgroup A. J. Virol..

[19] Lee H.J., Lee K.Y., Park Y.H., Choi H.J., Yao Y., Nair V., Han J.Y. (2017). Acquisition of resistance to avian leukosis virus subgroup B through mutations on tvb cysteine-rich domains in DF-1 chicken fibroblasts. Vet. Res..

[20] Lee H.J., Lee K.Y., Jung K.M., Park K.J., Lee K.O., Suh J.Y., Yao Y., Nair V., Han J.Y. (2017). Precise gene editing of chicken Na+/H+ exchange type 1 (chNHE1) confers resistance to avian leukosis virus subgroup J (ALV-J). Dev. Comp. Immunol..

[21] Ran F.A., Hsu P.D., Wright J., Agarwala V., Scott D.A., Zhang F. (2013). Genome engineering using the CRISPR-Cas9 system. Nat. Protoc..

[22] Hsu P.D., Scott D.A., Weinstein J.A., Ran F.A., Konermann S., Agarwala V., Li Y., Fine E.J., Wu X., Shalem O. (2013). DNA targeting specificity of RNA-guided Cas9 nucleases. Nat. Biotechnol..

[23] Himly M., Foster D.N., Bottoli I., Iacovoni J.S., Vogt P.K. (1998). The DF-1 chicken fibroblast cell line: Transformation induced by diverse oncogenes and cell death resulting from infection by avian leukosis viruses. Virology.

[24] Federspiel M.J., Hughes S.H. (1997). Retroviral gene delivery. Methods Cell Biol..

[25] Schusser B., Collarini E.J., Yi H., Izquierdo S.M., Fesler J., Pedersen D., Klasing K.C., Kaspers B., Harriman W.D., van de Lavoir M.C. (2013). Immunoglobulin knockout chickens via efficient homologous recombination in primordial germ cells. Proc. Natl. Acad. Sci. USA.

[26] Trefil P., Aumann D., Koslová A., Mucksová J., Benešová B., Kalina J., Wurmser C., Fries R., Elleder D., Schusser B. (2017). Male fertility restored by transplanting primordial germ cells into testes: A new way towards efficient transgenesis in chicken. Sci. Rep..

[27] Whitworth K.M., Rowland R.R., Ewen C.L., Trible B.R., Kerrigan M.A., Cino-Ozuna A.G., Samuel M.S., Lightner J.E., McLaren D.G., Mileham A.J. (2016). Gene-edited pigs are protected from porcine reproductive and respiratory syndrome virus. Nat. Biotechnol..

[28] Boddicker N.J., Bjorkquist A., Rowland R.R., Lunney J.K., Reecy J.M., Dekkers J.C. (2014). Genome-wide association and genomic prediction for host response to porcine reproductive and respiratory syndrome virus infection. Genet. Sel. Evol..

[29] Counillon L., Bouret Y., Marchiq I., Pouysségur J. (2016). Na(+)/H(+) antiporter (NHE1) and lactate/H(+) symporters (MCTs) in pH homeostasis and cancer metabolism. Biochim. Biophys. Acta.

[30] Denker S.P., Barber D.L. (2002). Cell migration requires both ion translocation and cytoskeletal anchoring by the Na-H exchanger NHE1. J. Cell Biol..

[31] Cardone R.A., Casavola V., Reshkin S.J. (2005). The role of disturbed pH dynamics and the Na+/H+ exchanger in metastasis. Nat. Rev. Cancer.

[32] Bourguignon L.Y., Singleton P.A., Diedrich F., Stern R., Gilad E. (2004). CD44 interaction with Na+-H+ exchanger (NHE1) creates acidic microenvironments leading to hyaluronidase-2 and cathepsin B activation and breast tumor cell invasion. J. Biol. Chem..

[33] Shrode L.D., Gan B.S., D’Souza S.J., Orlowski J., Grinstein S. (1998). Topological analysis of NHE1, the ubiquitous Na+/H+ exchanger using chymotryptic cleavage. Am. J. Physiol..

[34] Counillon L., Pouysségur J., Reithmeier R.A. (1994). The Na+/H+ exchanger NHE-1 possesses N- and O-linked glycosylation restricted to the first N-terminal extracellular domain. Biochemistry.

[35] Guan X., Zhang Y., Yu M., Ren C., Gao Y., Yun B., Liu Y., Wang Y., Qi X., Liu C. (2018). Residues 28 to 39 of the Extracellular Loop 1 of Chicken Na+/H+ Exchanger Type I Mediate Cell Binding and Entry of Subgroup J Avian Leukosis Virus. J. Virol..

